# Understanding Offending Patterns of Intimate Partner Violence: They Are Not All the Same

**DOI:** 10.1177/10778012251356949

**Published:** 2025-07-16

**Authors:** Christine T. Carney, Mark R. Kebbell, Li Eriksson, Regan M. Carr

**Affiliations:** 1 Griffith University, Mount Gravatt Campus, Mount Gravatt, QLD, Australia; 2 1969 Queensland University of Technology, Brisbane, QLD, Australia

**Keywords:** intimate partner violence, typology, cluster analysis, offenders

## Abstract

Intimate partner violence offenders may have a history of committing other offenses within and outside of the relationship context. This study used a police dataset of male and female offenders (*N* = 1,189), and their offenses recorded as solved between 2009 and 2019. Hierarchical cluster analyses and *K*-means focused on diversity in offending through a person-centered approach, establishing six cluster solutions for males and four cluster solutions for females. Male typologies included “low-level offenders,” “escalating intimate partner violence offenders,” “anti-social offenders,” “increasingly prolific violent offenders,” “escalating prolific generalist offenders,” and lastly, “de-escalating prolific offenders.” Similar typologies were evident across the female cohort with a “low-level female offender” typology, a “low-level escalating anti-social” female offender typology, an “intimate partner violence and regulatory offense female offender,” and lastly, “prolific violent and anti-social female offender” typology. Male and female offender typologies varied significantly across several variables. Similarities were identified across gender and typologies, alongside similarities and differences based on the time period of offending (either pre-2013, 2013, or post-2013). The implications of this study involve increased understanding of how police administrative data can be used to identify differences across offending based on sex and offense types and tailor responses accordingly.

An increasing focus on gendered violence across the Western world has seen billions of dollars dedicated to responding to domestic, family and sexual violence (Aridi et al., 2022; Commonwealth of Australia, 2022; Mennicke, 2019; Queensland Government, 2023; Scottish Government, 2023; Sparks, 2022; The White House, 2023). Much of the focus has been on legislative amendments, community awareness campaigns, and increased resources for policing and criminal justice agencies (Durfee & Goodmark, 2020; Hoppe et al., 2020; Mignon & Holmes, 1995; Queensland Government, 2023; Scottish Government, 2023). Intervention programs for male intimate partner violence (IPV) offenders^1^ have been developed to respond, although they often take a one-size-fits-all approach (Blatch et al., 2020; Sullivan, 2006; Whitaker & Lutzker, 2009; Women’s Safety and Justice Taskforce, 2021). Despite studies highlighting variation in offending behaviors (Alexander & Johnson, 2023; Boxall et al., 2015; Carney et al., 2023; Coleman et al., 2018; Francis et al., 2004; Holtzworth-Munroe & Meehan, 2004; Holtzworth-Munroe & Stuart, 1994; Johnson, 1995), much of the progress outside of academia to date has bypassed typology research in favor of a more homogenous approach (Gadd & Corr, 2017; Women’s Safety and Justice Taskforce, 2021). To address this, the present study aims to strengthen understanding and identification of diversity in offending patterns for both males and females recorded as IPV offenders in police administrative data from Queensland, Australia.

## IPV Typologies—Setting the Scene

Typology research is useful for understanding variations in the types of personal characteristics/traits, behaviors, modus operandi, and risks posed by offenders. Within IPV literature, typologies can help strengthen identification of, and response to subtypes of offenders who pose different risks, are motivated by different factors, and use different forms of abuse (Holtzworth-Munroe & Stuart, 1994; Johnson, 1995; Mennicke, 2016; Stark, 2007). Key scholars in the field of IPV typologies include Holtzworth-Munroe and Stuart (1994) and Johnson (1995). Given the extensive reference to their works throughout the literature, the typologies of Holtzworth-Munroe and Stuart (1994) and Johnson (1995) will be discussed in some detail, with brief examples of additional works that have drawn from existing typologies included.

Holtzworth-Munroe and Stuart (1994) conducted a review of previously developed typologies through an examination of typology literature involving 15 batterer typologies. These typologies were developed through either deductive or inductive methods, with samples derived from clinical observations, interviews with abused women, prison records, or depositions of males arrested for IPV (Holtzworth-Munroe & Stuart, 1994). From this review, three key descriptive dimensions were identified: (a) severity of marital violence, (b) generality of violence, and (c) psychopathology or personality disorders.

Later work validated the three original typologies and identified a fourth—low-level antisocial (Holtzworth-Munroe et al., 2000). Typology stability was confirmed at three different time points (over 3 years) using the three original dimensions of marital violence, general violence, and psychopathology. Replicability of these typologies across multiple studies using clinical and community samples, as well as similar studies to those of Holtzworth-Munroe and Stuart (1994), confirmed that IPV offenders were not all the same (Bland & Ariel, 2020; Eckhardt et al., 2008). Although limited, research examining female offenders also showed support for the typologies identified by Holtzworth-Munroe and Stuart (1994).

Whereas Holtzworth-Munroe and Stuart (1994) developed their typologies from an extensive review of existing typology literature, Johnson (1995) aimed to explain variance in the types of offenders seen in clinical/shelter samples and those found in community and national survey samples. The intent of that study was to provide support for the hypotheses that two distinct phenomena existed and could explain the contention regarding gender symmetry/asymmetry (Johnson, 1995). In his original study, Johnson (1995) identified two distinct types of violence—common couple violence perpetrated fairly equally by males and females, and patriarchal terrorism perpetrated mostly by men. Further exploration of IPV identified a further two types of offender, violent resistance (often used by women in response to experiencing patriarchal (later termed intimate) terrorism, and mutual violent control (where males and females both attempted to gain general control in the relationship) (Johnson, 2008). Similar typologies were identified through later studies and included unidirectional control (where one partner uses high levels of control but no violence), bidirectional control (where both partners use high control, no violence) and control resistance (where both partners are controlling and one partner is also violent) (Mennicke, 2019).

## Sex Differences in Offending

A systematic review of Australian quantitative studies (*n* = 39) identified the individual characteristics of IPV offenders, the proportion with prior offending histories, and prevalence of reoffending among this cohort (Hulme et al., 2019). Most of the studies included in the systematic review involved the use of police or courts administrative datasets (Hulme et al., 2019). The systematic review found that men comprised the majority of offenders (*Mdn* = 83%), and that around half of the women identified as offenders had also been recorded as a victim (Hulme et al., 2019). The findings of Francis et al. (2004), and Hulme et al. (2019) showed similarities in terms of criminal behaviors by age group.

Further studies exploring differences in offending have also identified specialist and generalist offenders (Babcock et al., 2003; Mazerolle & McPhedran, 2018; Piquero et al., 2006; Weatherburn & Rahman, 2018). For example, a recent study exploring degrees of specialization among female university students (*n* = 185) self-reporting either IPV or general violence found two potential models of offending (Wolbers & Ackerman, 2020). “Generalists” were most likely to commit property offenses, non-domestic violence offenses, and drug offenses. The second “IPV specialists” were least likely to commit general violence, property, or drug offenses compared to the other groups, although they did commit some non-domestic violence offenses. Wolbers and Ackerman’s (2020) study found older women were more likely to specialize when compared with younger participants from the sample. It is important to acknowledge that this study was focused on a sub-set of the female population and further exploration is required to understand whether these findings are generalizable to the broader population. Findings from this study differ from previous research examining diversity in offending (Johnson, 2008) in that self-defense does not appear to be a primary explanation for female IPV offending (Wolbers & Ackerman, 2020).

Analysis of data from the Spouse Assault Replication Program (SARP) examined whether male domestic violence offenders specialized in domestic violence or committed general violence as well as domestic violence (Piquero et al., 2006). For specialist offenders, a different approach would be required based on underlying factors influencing the offending behavior. For example, some IPV offenders are highly controlled in their use of both violent and nonviolent tactics, suggesting other factors may be relevant when responding to these offenders (Stark, 2007).

Results of the SARP in one study location found that 17.1% of suspects had at least one previous arrest, the majority (77.5%) of which were for nonviolent offenses (Piquero et al., 2006). A small portion (12.6%) had been arrested for violent offenses only. The majority of domestic violence offenders identified in the study were generalist in their offending, with some escalating and others de-escalating in their use of violence over time (Piquero et al., 2006). Offenders who did escalate tended to be the most severely violent initially and more likely to continue over time (Piquero et al., 2006). This suggests greater exploration of offending patterns is required to better understand how to respond to diversity in offending. It also suggests that by reviewing offending patterns over time, it may provide greater understanding of whether offenders continue to offend to the same extent or reduce offending over time.

## A Discussion on Typology Methods Used to Understand Offenders and Offending

A systematic review of clustering and classification studies (*n* = 34) found variation in statistical methods and results across the typology literature (Alexander & Johnson, 2023). The most widely used methods included hierarchical cluster analysis (HCA; Alexander & Johnson, 2023; Dutton et al., 2005; Holtzworth-Munroe et al., 2000; Johnson, 2006), latent class analysis (LCA; McKay et al., 2022), and latent profile analysis (LPA; Messing et al., 2016). Most of the included studies drew from survey or interview data, alongside measures derived from the Conflict Tactics Scale (CTS) and CTS2, and/or psychosocial assessments (*n* = 30) (Alexander & Johnson, 2023). Lesser used methods have also been discussed across the literature, including person-specific approaches involving dynamic factor analyses and principal axis factor analyses (oblimin) (Howard & Hoffman, 2017).

Person-centered analyses are useful for exploring subpopulations with shared attributes and for explaining group differences (Laursen & Hoff, 2006; Woo et al., 2024). Much of the discussion on person-centered methods identified through the literature has originated from the fields of psychology and organizational behavior, rather than from the field of criminology (Woo et al., 2024). Methods used for person-centered approaches may also be used for variable-centered approaches that are used to describe associations between the variables of interest (Laursen & Hoff, 2006; Muthén & Muthén, 2000). Some of these methods classify individuals across scores on a set of variables and can draw on cluster analysis, LCA, and LPA, factor mixture analysis, and mixture regression analysis (Woo et al., 2024). Two additional approaches are also useful when exploring subpopulations based on temporal fluctuations—growth mixture modeling and latent transition analysis (Woo et al., 2024). The most widely used methods for person-centered analyses appear to be HCA (using single/complete linkage or Ward's minimum variance), non-HCA (with *K*-means and *K*-medoids), or a mix of both (Woo et al., 2024).

Both LCA and LPA are popular methods in person-centered analyses but are somewhat constrained by the exploratory nature of these methods (Woo et al., 2024). Person-centered approaches such as those discussed analyze individual-level data to understand how subpopulations differ based on the configuration of variables included in the analyses (Woo et al., 2024). Limitations of the LCA and LPA methods include possible restriction of the potential number of solutions, risk of biased results because of overestimating the true number of latent classes evident in the dataset or because of inaccurate parameter estimates (Woo et al., 2024). Hierarchical and non-hierarchical cluster analyses using *K*-means are useful in clustering like objects together but use a “hard pairing” of cases meaning objects cannot move from one cluster to another once placed initially (Maharaj et al., 2019).

Person-specific approaches can be beneficial as these approaches focus on the unique characteristics or behaviors of a single individual, drawing from both qualitative and quantitative data with multiple variables incorporated into the analyses (Woo et al., 2024). Additional person-specific methods to those mentioned above include dynamic factor analysis, state-space modeling, and P-technique factor analysis (Howard & Hoffman, 2017; Woo et al., 2024). Factor analyses (principal component analysis [PCA] using SPSS) have been applied to official arrest records of a Danish birth cohort (*n* = 28,879) to identify subtypes (Collins et al., 1983). Exploratory factor analysis (EFA) and confirmatory factor analysis (CFA) have been used to understand the relationship between latent variables (factors) and observed indicators (e.g., identifying levels of depression based on multiple survey responses) (Roos & Bauldry, 2022). CFA has been used to understand the dimensions involved in sex offender offending patterns (*n* = 388) based on semi-structured interviews (Lussier et al., 2005). PCA has been used to categorize large-scale crime data at a country and provincial level of analysis (Shehzadi et al., 2021). This suggests various methodologies are useful for establishing typologies of offenders and offending patterns. Both EFA and HCA were used for this study, but due to page limits, only HCA is reported.

## The Current Study

The current study uses Australian police data to explore the patterns of offending evident in a sample of males and females recorded as IPV offenders (*N* = 1,189). The dataset includes all offense categories recorded by police based on the Australian and New Zealand Standard Offence Classification (ANZSOC). An additional offense category of IPV was created and included in the analyses. This is because to date, IP-related matters do not have a ANZSOC classification. Instead, the policing agency records additional information noting whether an ANZSOC offense was related to IPV within the offense description field in the records management system. For the purpose of this study, IPV-related offenses include offenses that have been recorded as contravene domestic violence, a police application, a private application, or a domestic violence—other action offense. Analyzing this full range of offense classifications is hoped to provide greater depth in understanding of the offending patterns of males and females recorded as IPV offenders. It is hoped that this will translate in practice to providing more targeted interventions for offenders. For example, if results were to include an IPV specialist typology, it may suggest more punitive responses based on level of violent offending identified, alongside cognitive behavioral therapy to understand the drivers of IPV. Alternatively, a therapeutic response may be more suited to generalist offenders with excessive drug- or alcohol-related offenses recorded. Offending histories have been categorized into three time periods for the purpose of this study—pre-2013 offending, offenses recorded in 2013, and offenses recorded post-2013. This use of time periods is hoped to provide greater context to understanding of offending patterns over time. Identification of offender typologies was achieved by answering the following research questions using HCA and the most useful analytical tool:

Research Question 1: Do distinct offending patterns/types exist for IPV offenders, and if so, what are they?
Research Question 2: Do overlaps exist in the types of clusters seen for men and women who commit IPV?

## Method

### Sample

Ethics approval was granted by a University Human Research Ethics Committee and a Police Service Research Committee. The sample was drawn from police administrative data extracted from the Records Management System that holds information on all matters referred to or dealt with by the police. The data for this study included offense histories drawn from a random sample of males and females who had been recorded as IPV offenders in 2013 (referred to as the index offense). Each offenders’ criminal histories were then extracted, with only offenses recorded between 2009 and 2017 included in the final sample. This was intentional to ensure that each individual's offending histories were restricted to 4 years pre-2013 and 4 years post-2013. The intent was to account for older offenders who may have had far more extensive histories that younger offenders in the sample. Only offenses recorded as solved were included in the analyses. The original sample included individuals recorded as an offender (*n* = 1,169) or both an offender and victim (*n* = 128) in the index IPV offense. Individuals recorded as both an offender and victim had been issued with what is termed a cross-order. Cross-orders are issued when police determine that both parties are deemed in need of protection from one another and/or it is too difficult to determine the primary offender.

Given that this study is related to offending behaviors, those individuals subject to cross-orders have been included in the analyses. Ages in the dataset were recorded as an age range. Given the focus on adults, anyone recorded as aged under 20 years was removed from the analyses. Criminal offense histories were provided for offenders and included traffic/regulatory offenses and criminal offenses. Records categorized as street checks (*n* = 5,793) were removed from analysis as these do not relate to offenses per se and do not record offender/victim status. Only offenses recorded against male and female offenders at the index offense and recorded between 2009 and 2017 were included in the analyses (*n* = 22,560). Of the total offenders recorded during the index offense, 87 offenders had no offenses recorded outside of the index offense. The final sample comprised offenders at the index offense (*n* = 1,189) and included males (*n* = 997) and females (*n* = 192).

The analyses for this study drew on multiple sets of data provided by the Queensland Police Service. Data included a random sample of index IPV offenses recorded by police throughout 2013. The reason for selecting an index offense was to understand offending patterns and diversity of people recorded specifically as an IPV offender. For the purpose of this study, only offenders from the index offense were included in further analyses. Once the index offenders were identified, a series of merges were undertaken using OpenRefine (a free open-access software package used to cleanse data) and Excel.

### Measures

#### Dependent Variables

To examine offending diversity, 62 count variables were included in the analyses. This included 19 variables based on the ANZSOC used for recording the administrative data for this dataset. Each offense falls under an overarching offense type such as homicide and related offenses, sexual assault and related offenses, and so on (Australian Bureau of Statistics, 2023). Further variables of total offenses recorded in pre-2013, 2013, and post-2013 were also included. A variable “IPV-related offenses” was created as IPV is not currently recorded as a separate offense in the ANZSOC. The IPV-related offenses variable includes the following: Domestic Violence—Police Application, Domestic Violence—Private Application, Domestic Violence (Contravene DFVPA), and Domestic Violence—Other. These same variables were reproduced for each time frame (pre-2013, in 2013, post-2013). Count variables ranged from 0 (did not commit offense) to 118 (the highest number of offenses). One dichotomous variable (1 = *yes*; 0 = *no*) was later included to identify whether statistically significant differences were evident in the offending patterns of males and females based on offenses committed within and outside of the relationship context.

#### Independent Variables

Independent dichotomous variables of sex (male/female) were included alongside categorical variables for age range of offenders: 20–29 years (1), 30–39 years (2), 40–49 years (3), 50–59 years (4), 60+ years (5).

#### Analysis

Frequencies and descriptive analyses were conducted to identify sample characteristics and perform tests of normality. Chi-square test of independence was conducted to explore the relationship between age range and sex. The results were not statistically significant, χ^2^(4) = 6.67, *p* = .155. An exploration of the diversity in the offending patterns of males and females recorded as IPV offenders in the 2013 index domestic violence offense was conducted using HCA.

HCA was performed using Ward's linkage method based on squared Euclidean distances, followed by *K*-means analyses. These methods were chosen as they are some of the most popular methods used for analyzing person-centric variables (Alexander & Johnson, 2023; Clatworthy et al., 2005). While similar methods have been used previously (such as LCA or LPA), HCA was deemed suitable because of the ability to subjectively select the number of clusters included. This has been done purposely to help identify offenders with extreme outliers and those with significantly less offending than the sample.

## Results

### Question 1: Do Distinct Offending Patterns/Types Exist Across the Sample, and If So, What Are They?

#### Male Offenders

HCA with Ward's linkage and squared Euclidean distances was conducted using SPSS version 29. Analysis involved 62 count variables of recorded crimes based on ANZSOC crime classifications. The agglomeration schedule and dendrogram suggested a six-cluster solution. The cluster solution was reached in 21 iterations with a minimum distance between centers at 56.939, suggesting good spacing between clusters. *K*-means cluster analyses were then performed to confirm the solution with variations including three, four, five, six, and seven clusters examined, with six clusters maintained. Distances between cluster centers ranged from 14.822 to 105.559. Clusters 1 and 2 demonstrated large distances between each other (105.559), indicating distinct differences when compared to the other clusters.

Cluster 3 showed moderate distance from all other clusters, whereas Clusters 4–6 had smaller distances between centers, suggesting similarities between these clusters. Despite three clusters having very small numbers, all six clusters were maintained to ensure that the nuances evident across the individuals in the sample were captured. To further test for differences across the clusters, one-way ANOVA was conducted. Results indicated statistically significant differences across the majority of variables tested. Caution should be taken when interpreting these results, given that clusters were chosen to maximize differences among the cases in different clusters.

Cluster 1 (*n* = 618), termed “low-level offenders,” comprised the largest of the male clusters. Low-level offenders were characterized by low-range offending across all variables for the three time periods (pre-2013, in 2013, and post-2013). Cluster 2 (*n* = 7) termed “increasingly prolific violent offenders” showed increased prevalence of offending across the three time periods, including offenses against the person (i.e., assault, sexual assault) and drug offenses. Cluster 3 (*n *=* *<5) termed “anti-social offenders” was the smallest of the clusters and characterized by limited offending pre-2013 prior to an escalation of offending, including IPV offenses in 2013. Cluster 3 was the only cluster to have no offending post-2013. There may be reasons for this not provided in the data, for example, incarceration, not coming to the attention of police, or not committing further offenses. Due to the very small number of offenders included in Clusters 2 and 3, caution must be taken when interpreting results.

Cluster 4 (*n* = 211), termed “escalating IPV” demonstrated relatively consistent offending across the three time periods. Escalating IPV offenders were characterized by escalating rates of IPV offending over the three time periods. While offending patterns for Cluster 4 were similar to those of Cluster 1, rates of offending were consistently higher in Cluster 4 across the three time periods. Cluster 5 (*n* = 32) termed “escalating prolific generalist offenders” was characterized by offending across most offense categories, although regulatory offenses were recorded at higher rates than offenses against the person.

Cluster 6 (*n* = 127), termed “de-escalating prolific offenders,” was characterized by high rates of offending pre-2013 across every single offense category, including the highest mean scores for homicide and related offenses (although still low), acts intended to cause injury, and domestic violence-related offenses. Offending declined in 2013 and post-2013 for offenders in Cluster 6. Differences across the six clusters were statistically significant for nearly all variables analyzed (see Table 1).

**Table 1.** Hierarchical Cluster Analysis—Male Index Offender (*n* = 997) Six-Cluster Solution.

Male clusters (*n* = 997)	Low-Level Offenders	Increasingly Prolific Violent Offenders	Anti-Social Offenders	Escalating IPV^a^	Escalating Prolific Generalist Offenders	De-Escalating Prolific Offenders	
Offenses	Cluster 1 *n* = 618 *M* (*SE*)	Cluster 2 *n* = 7 *M* (*SE*)	Cluster 3 *n* = <5^a^ *M* (*SE*)	Cluster 4 *n* = 211 *M* (*SE*)	Cluster 5 *n* = 32 *M* (*SE*)	Cluster 6 *n* = 127 *M* (*SE*)	*p*
Recorded offenses pre-2013							
01 Homicide and Related Offenses	0.00 (0.002)	0.00 (0.000)	0.00 (0.000)	0.00 (0.005)	0.00 (0.000)	0.01 (0.008)	.885
02 Acts Intended to Cause Injury	0.19 (0.021)	1.00 (0.488)	0.00 (0.000)	0.38 (0.046)	.50 (0.135)	1.22 (0.151)	<.001
03 Sexual Assault and Related Offenses	0.02 (0.007)	0.00 (0.000)	0.00 (0.000)	0.00 (0.005)	0.09 (0.052)	0.08 (0.029)	.004
04 Dangerous or Negligent Acts Endangering Persons	0.01 (0.004)	0.00 (0.000)	0.00 (0.000)	0.15 (0.063)	0.00 (0.000)	0.16 (0.041)	<.001
05 Abduction Harassment and Other Offenses Against the Person	0.01 (0.004)	0.00 (0.000)	0.00 (0.000)	0.00 (0.000)	0.00 (0.000)	0.01 (0.008)	.941
06 Robbery Extortion and Related	0.01 (0.003)	0.00 (0.000)	0.00 (0.000)	0.00 (0.000)	0.03 (0.031)	0.02 (0.014)	.156
07 Unlawful Entry With Intent Burglary Break and Enter	0.06 (0.015)	0.43 (0.297)	0.00 (0.000)	0.10 (0.025)	0.41 (0.126)	1.12 (0.328)	<.001
08 Theft and Related Offenses	0.11 (0.018)	2.43 (1.172)	2.00 (0.000)	0.40 (0.067)	1.28 (0.368)	1.80 (0.257)	<.001
09 Fraud Deception and Related Offenses	0.01 (0.005)	0.14 (0.143)	0.00 (0.000)	0.06 (0.019)	0.00 (0.000)	0.20 (0.050)	<.001
10 Illicit Drug Offenses	0.22 (0.026)	2.29 (1.643)	4.00 (0.000)	0.77 (0.095)	1.69 (0.413)	2.65 (0.274)	<.001
11 Prohibited and Regulated Weapons and Explosives Offenses	0.03 (0.007)	0.14 (0.143)	0.00 (0.000)	0.08 (0.019)	0.19 (0.083)	0.24 (0.053)	<.001
12 Property Damage and Environmental Pollution	0.09 (0.015)	0.29 (0.286)	0.00 (0.000)	0.26 (0.040)	0.88 (0.280)	1.06 (0.113)	<.001
13 Public Order Offenses	0.42 (0.035)	2.71 (0.778)	0.00 (0.000)	1.06 (0.092)	2.12 (0.332)	3.35 (0.315)	<.001
14 Traffic and Vehicle Regulatory Offenses	0.62 (0.044)	3.14 (1.079)	0.00 (0.000)	1.49 (0.133)	2.25 (0.416)	3.67 (0.290)	<.001
15 Offenses Against Justice Procedures	0.14 (0.020)	1.71 (1.128)	0.00 (0.000)	0.51 (0.070)	0.84 (0.250)	2.25 (0.214)	<.001
16 Miscellaneous Offenses	0.16 (0.019)	1.43 (0.812)	0.00 (0.000)	0.43 (0.056)	0.47 (0.180)	1.57 (0.210)	<.001
170 Police Use of Force OC	0.00 (0.002)	0.00 (0.000)	0.00 (0.000)	0.00 (0.000)	0.00 (0.000)	0.01 (0.008)	.742
171 Community By Laws	0.00 (0.002)	0.00 (0.000)	0.00 (0.000)	0.02 (0.010)	0.00 (0.000)	0.01 (0.008)	.125
300 Legislation Various	0.07 (0.012)	0.71 (0.286)	0.00 (0.000)	0.18 (.033)	0.34 (0.096)	0.63 (0.087)	<.001
DFV-related offenses	0.59 (0.052)	3.14 (1.580)	2.00 (0.000)	1.27 (0.130)	1.94 (0.679)	3.80 (0.348)	<.001
Total offenses	2.77 (0.135)	19.57 (4.95)	8.00 (0.000)	7.17 (0.318)	13.03 (1.68)	23.85 (0.795)	<.001
Recorded offenses in 2013							
01 Homicide and Related Offenses	0.00 (0.002)	0.00 (0.000)	0.00 (0.000)	0.00 (0.000)	0.00 (0.000)	0.00 (0.000)	.987
02 Acts Intended to Cause Injury	0.16 (0.017)	0.14 (0.143)	2.00 (0.000)	0.30 (0.037)	0.38 (0.133)	0.50 (0.067)	<.001
03 Sexual Assault and Related Offenses	0.00 (0.004)	0.00 (0.000)	0.00 (0.000)	0.00 (0.000)	0.00 (0.000)	0.04 (0.026)	.101
04 Dangerous or Negligent Acts Endangering Persons	0.02 (0.007)	0.00 (0.000)	0.00 (0.000)	0.04 (0.015)	0.13 (0.098)	0.05 (0.019)	.086
05 Abduction Harassment and Other Offenses Against the Person	0.00 (0.002)	0.00 (0.000)	0.00 (0.000)	0.00 (0.000)	0.00 (0.000)	0.00 (0.000)	.943
06 Robbery Extortion and Related	0.00 (0.005)	0.00 (0.000)	0.00 (0.000)	0.01 (0.008)	0.06 (0.043)	0.05 (0.025)	.046
07 Unlawful Entry With Intent Burglary Break and Enter	0.02 (0.006)	1.00 (0.577)	0.00 (0.000)	0.07 (0.021)	0.41 (0.155)	0.22 (0.068)	<.001
08 Theft and Related Offenses	0.06 (0.017)	2.71 (1.169)	2.00 (0.000)	0.28 (0.061)	1.38 (0.378)	0.56 (0.104)	<.001
09 Fraud Deception and Related Offenses	0.02 (0.007)	0.00 (0.000)	0.00 (0.000)	0.08 (0.040)	0.13 (0.074)	0.11 (0.048)	.058
10 Illicit Drug Offenses	0.21 (0.028)	2.57 (0.922)	18.00 (0.000)	0.67 (0.087)	1.34 (0.352)	0.58 (0.097)	<.001
11 Prohibited and Regulated Weapons and Explosives Offenses	0.02 (0.007)	0.14 (0.143)	0.00 (0.000)	0.03 (0.011)	0.19 (0.083)	0.07 (0.028)	<.001
12 Property Damage and Environmental Pollution	0.09 (0.012)	0.71 (0.360)	4.00 (0.000)	0.18 (0.031)	0.25 (0.119)	0.31 (0.063)	<.001
13 Public Order Offenses	0.22 (0.033)	1.00 (0.378)	2.00 (0.000)	0.67 (0.085)	1.09 (0.395)	0.86 (0.122)	<.001
14 Traffic and Vehicle Regulatory Offenses	0.31 (0.034)	0.57 (0.297)	0.00 (0.000)	0.53 (0.070)	1.47 (0.365)	0.83 (0.160)	<.001
15 Offenses Against Justice Procedures	0.14 (0.021)	0.43 (0.202)	2.00 (0.000)	.64 (.083)	1.16 (.318)	1.03 (.143)	<.001
16 Miscellaneous Offenses	0.09 (0.014)	0.86 (0.553)	2.00 (.000)	.32 (.052)	.44 (.148)	.50 (.074)	<.001
170 Police Use of Force OC	–	–	–	–	–	–	–
171 Community By Laws	0.00 (0.003)	0.00 (0.000)	0.00 (0.000)	0.00 (0.005)	0.00 (0.000)	0.00 (0.000)	.994
300 Legislation Various	0.05 (0.009)	0.29 (0.184)	0.00 (0.000)	0.08 (0.018)	0.34 (0.159)	0.11 (0.030)	<.001
DFV-related offenses	0.94 (0.052)	1.29 (0.360)	32.00 (0.000)	2.65 (0.188)	2.94 (0.655)	2.80 (0.331)	<.001
Total offenses	2.36 (0.127)	11.71 (2.73)	64.00 (0.000)	6.55 (0.363)	11.69 (1.89)	8.61 (0.687)	<.001
Recorded offenses post-2013							
01 Homicide and Related Offenses	0.00 (0.000)	0.00 (0.000)	0.00 (0.000)	0.00 (0.000)	0.00 (0.000)	0.01 (0.008)	.232
02 Acts Intended to Cause Injury	0.14 (0.018)	0.86 (0.404)	0.00 (0.000)	0.63 (0.065)	0.69 (0.176)	0.57 (0.071)	<.001
03 Sexual Assault and Related Offenses	0.02 (0.007)	1.14 (1.143)	0.00 (0.000)	0.01 (0.011)	0.00 (0.000)	0.03 (0.016)	<.001
04 Dangerous or Negligent Acts Endangering Persons	0.02 (0.006)	0.00 (0.000)	0.00 (0.000)	0.07 (0.020)	0.19 (0.095)	0.15 (0.067)	.001
05 Abduction Harassment and Other Offenses Against the Person	0.00 (0.004)	0.00 (0.000)	0.00 (0.000)	0.01 (0.011)	0.03 (0.031)	0.03 (0.016)	.285
06 Robbery Extortion and Related	0.01 (0.004)	0.29 (0.184)	0.00 (0.000)	0.05 (0.015)	0.13 (0.059)	0.04 (0.023)	<.001
07 Unlawful Entry With Intent Burglary Break and Enter	0.02 (0.007)	5.00 (2.182)	0.00 (0.000)	0.18 (0.034)	1.09 (0.243)	0.19 (0.046)	<.001
08 Theft and Related Offenses	0.07 (0.013)	23.00 (7.23)	0.00 (0.000)	0.84 (0.108)	9.84 (1.503)	0.76 (0.111)	<.001
09 Fraud Deception and Related Offenses	0.03 (0.010)	0.86 (0.404)	0.00 (0.000)	0.12 (0.030)	0.41 (0.224)	0.15 (0.053)	<.001
10 Illicit Drug Offenses	0.29 (0.036)	11.86 (3.70)	0.00 (0.000)	1.67 (0.140)	5.16 (0.707)	1.26 (0.195)	<.001
11 Prohibited and Regulated Weapons and Explosives Offenses	0.02 (0.005)	0.57 (0.297)	0.00 (0.000)	0.07 (0.020)	0.31 (0.095)	0.09 (0.027)	<.001
12 Property Damage and Environmental Pollution	0.06 (0.011)	0.71 (0.360)	0.00 (0.000)	0.44 (0.052)	1.69 (0.654)	0.35 (0.068)	<.001
13 Public Order Offenses	0.22 (0.021)	6.86 (2.650)	0.00 (0.000)	1.71 (0.136)	4.38 (0.636)	1.36 (0.150)	<.001
14 Traffic and Vehicle Regulatory Offenses	0.73 (0.049)	5.43 (1.730)	0.00 (0.000)	2.14 (0.162)	4.81 (0.761)	1.19 (0.174)	<.001
15 Offenses Against Justice Procedures	0.018 (0.453)	13.71 (5.77)	0.00 (0.000)	1.74 (0.128)	4.00 (0.584)	1.24 (0.140)	<.001
16 Miscellaneous Offenses	0.13 (0.020)	2.00 (0.577)	0.00 (0.000)	0.78 (0.084)	2.81 (0.593)	0.75 (0.119)	<.001
170 Police Use of Force OC	0.00 (0.000)	0.00 (0.000)	0.00 (0.000)	0.00 (0.000)	0.00 (0.000)	0.00 (0.000)	–
171 Community By Laws	0.00 (0.002)	0.00 (0.000)	0.00 (0.000)	0.03 (0.018)	0.00 (0.000)	0.00 (0.000)	.145
300 Legislation Various	0.06 (0.010)	2.86 (1.204)	0.00 (0.000)	0.37 (0.049)	0.72 (0.169)	0.42 (0.064)	<.001
DFV-related offenses	0.56 (0.043)	2.71 (1.085)	0.00 (0.000)	4.06 (0.268)	3.12 (0.711)	2.27 (0.249)	<.001
Total offenses	2.51 (0.109)	77.86 (4.38)	0.00 (0.000)	14.92 (0.397)	39.38 (1.52)	10.85 (0.621)	<.001

*Note. M* = mean; *SE* = standard error; IPV = intimate partner violence; DFV = domestic and family violence; OC = Oleoresin Capsicum (OC) spray.

a Where cluster numbers are small, <5 has been used to reduce potential for identification of individuals.

#### Female Offenders

HCA with Ward's linkage and squared Euclidean distances was conducted using SPSS version 29. The same 62 variables used to explore male offending were included in the female offender analyses. The agglomeration schedule and dendrogram suggested an eight-cluster solution initially. The cluster solution was reached in eight iterations with a minimum distance between centers at 41.376. Given that four of these clusters had two people or less, further refinement of clusters was conducted. This resulted in a final solution of four clusters (see Table 2) reached in 13 iterations with a minimum distance between initial centers of 67.528, suggesting good spacing between clusters.

**Table 2.** Hierarchical Cluster Analysis—Female Index Offender (*n* = 192) Four-Cluster Solution.

Female Offenders (*n* = 192)	IPV and Regulatory Offender	Low-Level Escalating Anti-Social Offender	Low-Level Offenders	Prolific Violent and Anti-Social Offender	
Offenses	Cluster 1 *n* = <5^a^ *M* (*SE*)	Cluster 2 *n* = 52 *M* (*SE*)	Cluster 3 *n* = 136 *M* (*SE*)	Cluster 4 *n* = <5^a^ *M*^b^	*p*
Recorded offenses pre-2013					
01 Homicide & Related Offenses	0.0 (0.0)	0.0 (0.0)	0.0 (0.0)	0.00	–
02 Acts Intended to Cause Injury	0.67 (0.67)	0.46 (0.12)	0.15 (0.05)	10.00	<.001
03 Sexual Assault & Related Offenses	0.0 (0.0)	0.0 (0.0)	0.0 (0.0)	4.00	–
04 Dangerous or Negligent Acts Endangering Persons	0.0 (0.0)	0.02 (0.02)	0.0 (0.0)	0.00	.444
05 Abduction Harassment & Other Offenses Against the Person	0.0 (0.0)	0.0 (0.0)	0.0 (0.0)	0.00	–
06 Robbery Extortion & Related	0.0 (0.0)	0.02 (0.02)	0.01 (0.01)	0.00	.908
07 Unlawful Entry Intent Burglary Break & Enter	0.0 (0.0)	0.15 (0.10)	0.01 (0.01)	0.00	.141
08 Theft & Related Offenses	0.33 (0.33)	0.96 (0.38)	0.023 (0.01)	10.00	<.001
09 Fraud Deception & Related Offenses	0.0 (0.0)	0.23 (0.10)	0.0 (0.0)	0.00	.006*
10 Illicit Drug Offenses	0.67 (0.67)	1.06 (0.26)	0.13 (0.04)	4.00	<.001
11 Prohibited & Regulated Weapons & Explosives	0.0 (0.0)	0.10 (0.04)	0.0 (0.0)	0.00	.003*
12 Property Damage & Environmental Pollution	0.67 (0.67)	0.44 (0.13)	0.08 (0.02)	12.00	<.001
13 Public Order Offenses	7.00 (0.58)	1.87 (0.28)	0.32 (0.07)	36.00	<.001
14 Traffic & Vehicle Regulatory Offenses	6.00 (5.03)	1.31 (0.28)	0.30 (0.07)	0.00	<.001
15 Offenses Against Justice Procedures	1.00 (0.58)	0.65 (0.17)	0.07 (0.03)	32.00	<.001
16 Miscellaneous Offenses	10.33 (5.78)	0.6 (0.17)	0.07 (0.03)	8.00	<.001
170 Police Use of Force OC	0.0 (0.0)	0.0 (0.0)	0.0 (0.0)	0.00	–
171 Community By Laws	0.0 (0.0)	0.02 (0.02)	0.0 (0.0)	0.00	.444
300 Legislation Various	0.33	0.12 (0.04)	0.08 (0.03)	0.00	.501
DFV-related offenses	20.33	3.29 (0.58)	0.6 (0.12)	2.00	<.001
Total offenses	47.33	11.29 (1.15)	1.83 (0.26)	118.00	<.001
Recorded offenses in 2013					
01 Homicide & Related Offenses	0.0 (0.0)	0.0 (0.0)	0.0 (0.0)	0.00	–
02 Acts Intended to Cause Injury	0.33 (0.33)	0.33 (0.09)	0.14 (0.03)	0.00	.130
03 Sexual Assault & Related Offenses	0.0 (0.0)	0.0 (0.0)	0.0 (0.0)	0.00	–
04 Dangerous or Negligent Acts Endangering Persons	0.0 (0.0)	0.06 (0.03)	0.01 (0.01)	0.00	.193
05 Abduction Harassment & Other Offenses Against the Person	0.0 (0.0)	0.00 (0.00)	0.0 (0.0)	0.00	–
06 Robbery Extortion & Related	0.0 (0.0)	0.00 (0.00)	0.0 (0.0)	0.00	–
07 Unlawful Entry Intent Burglary Break & Enter	0.0 (0.0)	0.13 (0.07)	0.01 (0.01)	0.00	.056
08 Theft & Related Offenses	0.0 (0.0)	0.29 (0.12)	0.04 (0.03)	0.00	.045*
09 Fraud Deception & Related Offenses	0.0 (0.0)	0.13 (0.12)	0.0 (0.0)	0.00	.313
10 Illicit Drug Offenses	0.67 (0.67)	0.52 (0.15)	0.07 (0.03)	0.00	<.001
11 Prohibited & Regulated Weapons & Explosives	0.0 (0.0)	0.04 (0.03)	0.0 (0.0)	0.00	.144
12 Property Damage & Environmental Pollution	0.0 (0.0)	0.25 (0.09)	0.04 (0.02)	0.00	.007*
13 Public Order Offenses	2.0 (1.15)	0.73 (0.16)	0.24 (0.07)	4.00	<.001
14 Traffic & Vehicle Regulatory Offenses	0.67 (0.67)	0.58 (0.19)	0.23 (0.09)	0.00	.259
15 Offenses Against Justice Procedures	1.67 (0.88)	0.79 (0.22)	0.07 (0.04)	0.00	<.001
16 Miscellaneous Offenses	0.34 (0.33)	0.33 (0.09)	0.08 (0.04)	0.00	.019*
170 Police Use of Force OC	–	–	–	–	–
171 Community By Laws	0.0 (0.0)	0.0 (0.0)	0.01 (0.01)	0.00	.939
300 Legislation Various	0.0 (0.0)	0.27 (0.16)	0.05 (0.03)	0.00	.278
DFV-related offenses	11.0 (1.73)	3.46 (0.76)	1.010 (0.16)	2.00	<.001
Total offenses	16.67 (4.37)	7.9 (1.09)	2.07 (0.32)	6.00	<.001
Recorded offenses post-2013					
01 Homicide & Related Offenses	0.0 (0.0)	0.0 (0.0)	0.0 (0.0)	0.00	–
02 Acts Intended to Cause Injury	0.34 (0.33)	0.69 (0.12)	0.04 (0.02)	6.00	<.001
03 Sexual Assault & Related Offenses	0.0 (0.0)	0.0 (0.0)	0.0 (0.0)	0.00	–
04 Dangerous or Negligent Acts Endangering Persons	0 (0.00)	0.08 (0.05)	0.01 (0.01)	0.00	.162
05 Abduction Harassment & Other Offenses Against the Person	0.0 (0.0)	0.0 (0.0)	0.0 (0.0)	0.00	–
06 Robbery Extortion & Related	0.0 (0.0)	0.02 (0.02)	0.0 (0.0)	0.00	.444
07 Unlawful Entry Intent Burglary Break & Enter	0.0 (0.0)	0.29 (0.20)	0.0 (0.0)	2.00	.007*
08 Theft & Related Offenses	0.0 (0.0)	1.38 (0.33)	0.08 (0.03)	18.00	<.001
09 Fraud Deception & Related Offenses	0.0 (0.0)	0.29 (0.10)	0.01 (0.01)	0.00	<.001
10 Illicit Drug Offenses	2.00 (2.0)	2.85 (0.59)	0.24 (0.08)	4.00	<.001
11 Prohibited & Regulated Weapons & Explosives	0.0 (0.0)	0.10 (0.05)	0.0 (0.0)	0.00	.020
12 Property Damage & Environmental Pollution	0.0 (0.0)	0.52 (0.12)	0.07 (0.03)	10.00	<.001
13 Public Order Offenses	3.00 (2.52)	2.02 (0.24)	0.27 (0.07)	46.00	<.001
14 Traffic & Vehicle Regulatory Offenses	0.00 (0.00)	2.13 (0.36)	0.30 (0.06)	0.00	<.001
15 Offenses Against Justice Procedures	1.00 (0.58)	2.21 (0.43)	0.07 (0.03)	10.00	<.001
16 Miscellaneous Offenses	4.00 (2.31)	1.12 (0.25)	0.15 (0.05)	6.00	<.001
170 Police Use of Force OC	0.0 (0.0)	0.0 (0.0)	0.0 (0.0)	0.00	–
171 Community By Laws	0.0 (0.0)	0.0 (0.0)	0.04 (0.03)	0.00	.896
300 Legislation Various	0.0 (0.0)	0.21 (0.09)	0.01 (0.01)	2.00	<.001
DFV-related offenses	10.67 (4.81)	5.19 (0.85)	1.11 (0.16)	4.00	<.001
Total offenses	21 (11.59)	19.10 (1.36)	2.40 (0.26)	108.00	<.001

*Note*. *M* = mean; *SE* = standard error; IPV = intimate partner violence; DFV = domestic and family violence; OC = Oleoresin Capsicum (OC) spray.

a Where cluster numbers are small, <5 has been used to reduce potential for identification of individuals. ^b^*SE* not calculated due to cell count less than 5.

*Levels are significant at the <.05 level.

*K*-means analyses were performed to assess the separation between the four cluster solution with the squared Euclidean distances between the cluster centroids ranging from 21.459 to 173.537 with the smallest distance (21.459) evident between Cluster 2 and Cluster 3. This indicated that Cluster 2 and Cluster 3 were more similar to each other when compared to the other clusters. The largest distance (173.537) was observed between Cluster 3 and Cluster 4, suggesting distinct differences between the two clusters. Clusters 1 and 2 were moderately distant (43.844), suggesting some level of separation between the clusters. In contrast, the distance between Cluster 1 and Cluster 4 (133.146) suggested a strong separation between the two clusters. To further test for differences between the clusters, one-way ANOVA was conducted. Results indicated statistically significant differences across several offense types and across the four clusters. Caution should be taken, however, in interpreting *F*-statistic results as clusters were chosen to maximize differences among the cases in different clusters.

Cluster 1 (*n *=* *<5) termed “IPV and regulatory offender” was characterized by regulatory offending and high levels of IPV across the three time periods. Female offenders in Cluster 1 were recorded against the highest numbers of domestic and family violence (DFV)-related offenses of all the clusters with rates statistically significantly different when compared to the remaining clusters (*p* < .001). Cluster 2 (*n* = 52), termed “low-level escalating anti-social offender,” was characterized by escalating IPV and regulatory offenses. Female offenders in Cluster 2 were recorded against drug offenses pre-2013, which decreased in 2013 before doubling post-2013. With the exception of DFV-related offenses in 2013, rates of offending across all other offenses appeared to be low.

Cluster 3 (*n* = 136) was the largest of the clusters but was least likely to be recorded against any offenses. Cluster 4 (*n* < 5), termed “prolific violent, anti-social offender,” was characterized by very high rates of offending when compared to all other clusters. This included serious violent offenses pre-2013 involving acts intended to cause injury, sexual assault and related offenses, and IPV. In 2013, a small number of offenses were recorded, including public order offenses that were statistically significantly different when compared to the other clusters (*p* < .001) along with DFV-related offenses. Cluster 4 was recorded against a very high volume of offenses when compared to the other clusters post-2013, which was statistically significantly different (*p* < .001). As with offenses recorded pre-2013, offending included acts intended to cause injury, DFV-related offenses, and a range of regulatory and criminal offenses.

### Question 2: Do Overlaps Exist in the Types of Clusters Seen for Men and Women?

Once cluster membership for male and female offenders was established, comparisons were conducted to determine whether similarities existed between the sexes. Low-level offenders were evident in both the male and female offender clusters, with offending characterized by very low rates of offenses across all ANZSOC classifications. Low-level offenders accounted for the largest portion of offenders overall (63.4%). Of the total female sample (*n* = 192), 70.8% were recorded as low-level offenders against offenses including assault, theft, illicit drugs, traffic offenses, regulatory offenses, and DFV-related offenses with very low means. Of the total male sample (*n* = 997), more than half (62.0%) were recorded in the low-level offending cluster. Low-level male offenders were recorded against similar offenses to females in the low-level cluster, with the addition of sexual assault related offenses. Based on the mean of each offense category, the level of offending across all ANZSOC categories was low in this cluster Table 3.

**Table 3.** Intimate Partner Violence Offender Typologies—A Comparison of the Holtzworth-Munroe and Stuart Typologies and Current Study Results.

Holtzworth-Munroe and Stuart (1994) and Holtzworth-Munroe and Meehan (2004)	Family-Only Batterer	Dysphoric/Borderline Batterer	Generally Violent Antisocial	Low-Level Antisocial
Definition	Least marital violence, least likely to be generally violent	Moderate to severe family violence, low-moderate general violence/criminality	Moderate to severe family violence, highest levels of general violence/criminality	Falls in between family only and generally violent/antisocial
Typologies based on HCA, K-means^a^	Escalating IPV males	Anti-social male offenders	Increasingly prolific violent males	Low-level male offenders
Definition	Low-level but escalating IPV, low-level general offending	Very high levels of IPV limited to one time period, low-level general crimes	Very high levels of general offending, moderate levels of IPV	Low-level general violent, anti-social, and IPV offending
Typologies based on HCA, K-means^a^		IPV and regulatory female offender	Escalating prolific generalist males	Low-level female offenders
Definition		High but declining levels of IPV and moderate levels of general anti-social violence	Moderate levels of general offending and IPV increasing	Consistently low-level anti-social and IPV offending
Typologies based on HCA, K-means^a^			De-escalating prolific males	Low-level escalating anti-social female offender
Definition			High to moderate levels of general violence and IPV	Low-level but escalating violence, anti-social, and IPV offending
Typologies based on HCA, K-means^a^			Prolific violent and anti-social females	
Definition			High levels of violence and anti-social behaviors	

*Note.* HCA = hierarchical cluster analysis; IPV = intimate partner violence.

a Typologies based on HCA and *K*-means based on police administrative offense data only. psychological/personality characteristics were not available for analyses.

Both male and female offender clusters identified “prolific offenders” although numbers for both sexes were small. Female “prolific offenders” comprised just 0.5% of the total female sample (*n* = 192), and males accounted for 0.7% of the total male sample (*n* = 997). Similarities across the sexes were also evident with “escalating offenses.” A larger number of female offenders were identified in escalating clusters, comprising 27.1% of the total female sample (*n* = 192). This differed from male offenders, with 3.2% of the total male sample identified in the escalating offense category. Differences also emerged between male and female offenders with male offending within the “DFV-related offenses” category. Roughly 1:5 male offenders of the total male sample were categorized into this group with offenses escalating over the three time periods. Female offenders were also identified as escalating in DFV-related offenses over the three time periods, although fewer females (1.6%) than males (21.6%) were included within these clusters.

## Discussion

This study explored the offending typologies of males and females randomly sampled from police administrative data and recorded against an index IPV offense in 2013. The first question explored whether distinct offending patterns/types existed for males and females. This question focused on male offenders and female offenders separately to understand and identify whether different types of male offenders existed and whether different types of female offenders existed. Statistically significant differences were evident across male offender typologies. For example, when examining differences in offending pre-2013, male offender typologies differed significantly across several variables, including assault, unlawful entry with intent, theft and related offenses, drug offenses, regulatory offenses, and DFV-related offenses. Similar patterns were also evident in 2013 and post-2013.

As with male offender typologies, those of females also identified statistically significant differences between female offender typologies. Statistically significant variables across clusters included assault, theft, drugs, regulatory offenses, and DFV-related offenses pre-2013. Fewer statistically significant differences were evident in 2013 with only a handful of variables, including drugs, property damage, and public order offenses, and DFV-related offenses statistically significantly different. Several variables were identified as statistically significantly different across female typologies post-2013. These included assault, theft and related offenses, regulatory offenses, miscellaneous offenses, and DFV-related offenses.

The second question focused on whether overlaps existed in the types of male and female offenders identified from question one. Similarities and differences were evident across the sexes with six male offender typologies and four female offender typologies identified. Two low-level female offender typologies and one low-level male typology were identified from the sample. These three typologies were characterized by low-level general violence, general anti-social behaviors (such as regulatory offenses) and low levels of IPV offending. While the male typology (low-level offenders) and one female typology (low-level offenders) were relatively steady in terms of the rate of offending, the low-level escalating anti-social female offenders were characterized by an escalation in overall offense rates between pre-2013 and post-2013 time periods. A second male typology, escalating IPV offenders, were characterized by low levels of general violence and escalating rates of IPV over the three time periods. Although similar to the low-level escalating anti-social females, males were recorded against lower rates of offenses when compared to females in the similar typology. This included less anti-social type offending behaviors for males when compared to the females.

Two typologies were characterized by high to very high levels of IPV, and low to moderate levels of general violence and anti-social behaviors. These typologies included anti-social male offenders recorded against high levels of IPV for one time period, and low-level general violence, and IPV and regulatory female offenders. The females were characterized by high but declining levels of IPV and moderate levels of general and anti-social violence.

Three generally violent male typologies and one female typology were also identified from the HCA. The three male typologies were distinguished by prevalence of violence with one group, “increasingly prolific violent males” characterized by very high levels of violence that increased over time, an “escalating prolific generalist male offenders” typology with moderate level of general offending and increasing levels of IPV, and a “de-escalating prolific male offender” typology characterized by high levels of general violence and IPV across the three time periods. A “prolific violent and anti-social female” typology was evident and characterized by high levels of violence and anti-social behaviors.

What this study shows is that not all IPV offenders are the same, with anti-social behaviors and violence manifesting in many ways. As noted in prior research, understanding these types of differences in offending, as well as whether someone offends, is beneficial for responding to criminality (Eckhardt et al., 2008; Ruiz & González-Calderón, 2022). Understanding the types of offenders that exist, along with their offending behaviors are useful for a variety of reasons. For example, it can assist with assessing risk, understanding what may work in terms of response, and in understanding the level of harm that may be caused by particular offenders (Ruiz & González-Calderón, 2022). This study also demonstrates the difference in the types of offender behaviors captured by police administrative datasets when compared to samples derived from general population samples, service providers, and clinical samples.

This study adds to gendered discussions on IPV (for example, Dobash et al., 1992; Johnson, 2006; Stark, 2007), by identifying that males and females recorded as IPV offenders are also recorded against similar offenses outside of the relationship. It has also demonstrated that males and females have both similarities and differences in terms of offending behaviors. At the same time, this study shows that males are far more likely to be recorded as offenders when compared to females—at least across police administrative data. Although not directly comparable due to differences in sample size and type, datasets and methodology used, the findings from this study show similarities to previous typology research such as that conducted by Holtzworth-Munroe et al. (2000); Holtzworth-Munroe and Stuart (1994) (see Table 4 for comparison).

**Table 4.** Alignment to Holtzworth-Munroe Typologies.

Holtzworth-Munroe and Stuart (1994) and Holtzworth-Munroe and Meehan (2004)	Family-Only Batterer	Dysphoric/Borderline Batterer	Generally Violent Antisocial	Low-Level Antisocial
Definition	Least severe marital violence, least likely to be generally violent	Moderate to high family violence, low-moderate general violence/criminality	Moderate to high family violence, high general violence/criminality	Falls in between family only and generally violent/antisocial
Typology based on factor analysis	Domestically violent (M)	Declining anti-social (F)	Consistently violent anti-social (M)	Drug anti-social (M and F)
	One-off offender (F)	Violent anti-social (F)	Emerging anti-social (M)	Risky driver (M and F)

*Note*. M = male; F = female.

### Limitations

Caution has been noted across typology literature when establishing categorical or typological approaches to offenders and offending behaviors, particularly regarding IPV (Alexander & Johnson, 2023; Holtzworth-Munroe & Meehan, 2004). This caution is partly due to variations across methods, datasets, study foci, and types of variables included in the analyses (Alexander & Johnson, 2023; Johnson, 1995, 2008). While HCA is a popular method for identifying types or patterns in study samples, like all forms of analyses, it is subject to constraints (Fox & Escue, 2022). These constraints include the subjective nature of clustering reliant upon the researcher's interpretation of cluster numbers from the Dendrogram output and Agglomeration schedule. This subjectiveness has been addressed somewhat through the use of additional statistical analyses, such as the *K*-means test and testing multiple cluster numbers prior to settling on the final cluster solution.

The current study aimed to include as much information on the dataset and methodology used so as to increase understanding of how the typologies were identified and interpreted. Limitations of the dataset included a lack of information on offender ethnicity, Australian Aboriginal and Torres Strait Islander status, geographic location, and intersectional information that may provide greater understanding of the offenders in the sample (i.e., disability, psychopathology, actual age, vulnerabilities). The study intentionally excluded offenders aged under 20 years for two main reasons. Firstly, age was only provided as an age range (e.g., 14–19 years) and as such the actual age was not able to be calculated. Secondly, as the study aimed to examine offending patterns over time, it would be unlikely that people aged under 20 years would have sufficient offenses recorded due to differences in how juvenile offenders (below 16 years) and adult offenders are recorded.

As with any official data, police administrative data is reliant upon crimes coming to the attention of police. The dark figure of crime is well known across the literature, especially for personal crimes such as IPV and sexual violence (Buil-Gil et al., 2020, 2021). Because of this, it is likely far more offenses have occurred than what have been included in the dataset. Police administrative data is also dependent upon the quality of information entered into the police records and information management system. It is possible that not all offenses have been recorded against each offender—particularly when multiple types of offenses have occurred within a single IPV incident.

Given this caution should be taken when interpreting results without taking these limitations into account. The data provided for this study excludes incarceration histories of the offenders in the sample. It is possible for those offenders with no or limited offending within one or more time periods, to have been incarcerated or under parole conditions and under greater surveillance, thus limiting opportunities for offending. These limitations reduce the overall ability to fully understand the types of offenders committing IPV and their offending patterns within and outside of the relationship context. But typologies do help understand and guide policy and practice toward areas in need of further enhancement such as data collection, collation, and analysis.

### Implications

The typologies developed in the current study were statistically derived and based on multiple methods to reduce the subjective nature of HCA. Use of administrative data limited the ability to gain in-depth, nuanced understandings of static and dynamic factors influencing or driving criminality of the sample. Despite this, police administrative data is useful for identifying the types of people and matters that come to police attention. The information captured by police at the scene of a crime/incident and recorded in the information management system is sometimes all police have to go on when determining an appropriate response. While limitations of the data were noted previously, deriving typologies from police administrative data provides potential for the same methods to be replicated across police departments to identify typologies relevant to the local context.

Typological literature has highlighted similarities and differences across cohorts. This does not necessarily mean some typologies are correct and others wrong. What this means is that not all offending and offenders are the same. Rather, nuances exist and should be used to help identify appropriate responses to each individual case. Identifying typologies of offending for males and females from police administrative data could support greater opportunities for appropriate intervention and increase understanding and awareness of the nuances involved in what is a very complex issue.

## Notes

1. The term offender is used throughout this paper to refer to a person who has been recorded as an intimate partner violence perpetrator, a respondent to an intimate partner violence-related matter as well as a person who has been recorded against offenses outside of the relationship. Intimate partner violence and domestic violence are used interchangeably throughout this paper to refer to violence committed in the context of an intimate relationship such as married, de-facto, ex-partner or current partner.
